# American and German attitudes towards cow-calf separation on dairy farms

**DOI:** 10.1371/journal.pone.0174013

**Published:** 2017-03-16

**Authors:** Gesa Busch, Daniel M. Weary, Achim Spiller, Marina A. G. von Keyserlingk

**Affiliations:** 1 Animal Welfare Program, Faculty of Land and Food Systems, University of British Columbia, Vancouver, British Columbia, Canada; 2 Marketing for Food and Agricultural Products, Department of Agricultural Economics and Rural Development, Faculty of Agricultural Sciences, Georg-August-University Göttingen, Niedersachsen, Germany; Universidade do Porto Instituto de Biologia Molecular e Celular, PORTUGAL

## Abstract

Public concerns regarding the quality of life of farm animals are often focused on specific practices such as separating the cow and calf immediately after birth. The available scientific literature provides some evidence in support of this practice (including reduced acute responses to separation when it does occur), as well as evidence of disadvantages (such as increased risk of uterine disease in cows). The aim of this study is to systematically examine public views around this practice. Specifically, this study analyzes the views of American and German citizens to separation of cow and calf at birth using a quantitative segmentation approach. Although the majority of participants opposed early separation, a small proportion of our sample supported the practice. According to participants’ preference for early and later separation and their evaluation of different arguments for both practices, three clusters were identified. US participants were more likely to support early separation compared to German participants. The arguments presented for and against both practices caused different reactions in the three clusters, but did not appear to sway the opinions of most participants. The results show considerable opposition to the practice of early separation in large parts of the sample and suggest that the dairy industry should consider approaches to address this concern.

## Introduction

In recent years there has been increasing public concern regarding the quality of life of farm animals [1,2,3]. The majority of work to date addressing citizens’ evaluation of contentious farming practices has taken place in Europe but this type of work is now gaining traction in North America [4,5]. Primary public concerns are in relation to restriction of movement (e.g. sows in gestation stalls [6]; laying hens in cages [7]), painful procedures (e.g. dehorning of dairy calves [8] and castration of piglets [9]) or lack of natural behavior (e.g. dairy cows access to pasture [10]). Some people working within agriculture have argued that these criticisms are unwarranted given that the public lacks knowledge of farming practices; from this perspective, criticisms will wane if the public is educated about agricultural production methods and the context of their use [11]. Alternatively, critiques of animal agriculture may reflect a lack of shared values surrounding agricultural production [12], making educational efforts unlikely to succeed in heading off criticisms about husbandry practices [13].

Political reactions to these criticisms have varied with some countries introducing legislation specific to farm animal welfare (e.g. implementation of national laws prohibiting tail docking and teeth clipping in piglets in Sweden and Norway; [14]), while other countries have relied on industry led initiatives (e.g. Canada; [15]).

Compared to the pork and poultry industries, dairy farming has received less attention, perhaps because dairy has a more positive public image than other livestock industries [16,17,18,19]. Some have argued that this positive perception of the dairy industry is a direct consequence of dairy marketing strategies and the higher visibility of cows in the countryside through open barns and pasture access [20]. However, some practices in dairy farming are also being increasingly discussed in the public.

Separating the calf from the cow shortly after birth is a routine practice on dairy farms around the world. In brief, calves are separated from their dams within a few hours following birth and then housed in a separate location and fed artificially (with milk or milk replacer). Advocates of this practice argue that it prevents cow and calf from forming a strong bond, and thus reduces the stress associated with separation when it does occur. The results from a series of studies on the short-term acute affects support this claim (e.g. [21,22,23]). Other arguments in support of early separation include better supervision of calf colostrum and milk intake [24]. One economic argument frequently presented by farmers is that the practice facilitates harvesting the maximum amount of milk available for sale [25]. Moreover, currently used housing and husbandry systems are not designed for keeping cow-calf pairs, making a change in practice difficult. Others have also argued that immediate separation reduces the risk of disease transfer to the calf, including paratuberculosis [26].

Arguments for later separation emphasize allowing more natural living of cow and calf [25], including emotional benefits for both [27]. Furthermore, some research has shown biological benefits for calves, including better weight gains [28] and fewer bouts of diarrhea when separated later [21], as well as improved udder [29] and uterine health [24] for cows.

The topic of early cow-calf separation can evoke strong negative responses from the public. Walker et al. [30] reported that 90% of their sample believed that the separation of animal parent and offspring results in the animal grieving. From the public’s perspective, when they are made aware of this practice, early cow-calf separation appears to have little support from those not working in the dairy industry [13,31].

Citizen perceptions of animal welfare are more diverse than farmers [32], so it is likely that there is no unified ‘public opinion’ on animal welfare topics. Segmentation, a tool used in consumer research to detect target groups for special commercial products, may be useful to determine diverse opinions and for garnering insights on particularly contentious animal welfare concerns. For example, Vanhonacker and Verbeke [2] argue in favor of this approach in animal welfare research as drawing conclusions from sample means is likely to obscure important voices, including those very concerned and those that are unconcerned with the issue. Other work has also shown differences in segments according to consumer and citizen attitudes towards animal products or animal production systems in different countries (e.g. Brazil [33]; USA [5,34]; Belgium, Poland, Denmark and Germany [4]; Netherlands [35]; Belgium [36]). Te Velde et al. [32] also provided evidence in support of segmentation and argued that research into the values and norms of different groups within society is needed to build a robust societal contract between farmers and consumers.

We are unaware of any work that has addressed the topic of cow-calf separation using a quantitative approach. The available literature on contentious practices within the dairy industry has focused on the acceptance and evaluation of the whole dairy sector (e.g. [16,17,37]) or on qualitative approaches to particular practices (e.g. [38,39]). The current study addresses this gap by analyzing the distribution of citizen attitudes towards a contested production practice in dairy farming using a quantitative method. We focus on the common but contentious practice of cow-calf separation in the hours immediately following birth.

A qualitative study by Ventura et al. [31] used a convenience sample of North Americans to examine the views of people from inside and outside the dairy industry on the issue of separation of the dairy cow from her calf immediately following birth. These authors identified six primary themes in the qualitative responses provided by 163 participants in the study: cow and calf emotions, calf health, cow health and production, a natural life, dissatisfactions with industry motivations and the changeability of dairy farming systems. These six themes (and the related arguments) were used to build the survey for this study. Specifically, we addressed the following objectives: 1) When do citizens think that cows and calves should be separated in dairy farming, 2) Can citizens be segmented according to their attitudes towards the different arguments of cow-calf separation and if so, what variables can be used to profile these groups and, 3) Does the provision of information in the form of arguments about the issue lead to a shift in participants’ opinion? The results are hoped to help those working within the dairy industry and policy makers to develop socially sustainable dairy farming practices.

Furthermore, values are influenced by cultural norms within societies [16] thus differences between countries may exist if values differ [15]. Vanhonacker and Verbeke [2] suggested that “nationality is not a key-characteristic for pro welfare behavior”, but little work to date has examined responses to welfare issues in residents of different countries, and none has done so in relation to early cow-calf separation. Thus our objective 4) was to draw upon an American and German sample to assess whether participants vary from these two countries.

## Materials and methods

We conducted an online survey with US and German citizens. The Behavioral Research Ethics Board at the University of British Columbia, Canada approved the study. Participants were explicitly informed that all data is treated confidentially and that only anonymous questionnaires were used. Participants had to consent to take part in the study and could withdraw consent at any point by simply closing their Internet browser.

### Survey design

A quantitative questionnaire was designed initially in the English language. For the German sample, the questionnaire was translated into German. To ensure the quality and validity of the translation it was back-translated into English by a German speaking researcher familiar with dairy farming but not involved in the study, and then adjusted as needed.

The main part of the survey contained 22 arguments that were based on the six themes (and 12 subthemes) identified by Ventura et al. [31] (see Table 1). These arguments provide information about the topic of separation in order to test for effects on opinion about the issue. For the current study we developed two arguments for each of the subthemes based on the original quotes from Ventura et al. [31]. For the subthemes “Dishonesty/Wrong focus” and “Profit motives” (part of the theme “Dissatisfaction with industry motives”) only one item was used to avoid redundancy given that similar arguments were presented. To reduce acquiescence bias in surveys [40,41] one of the arguments for each subtheme was framed positively while the other was framed negatively. Examples for the development of questions are given in Table 1. Agreement to the statements was tested using 7-point Likert-scales ranging from -3 =“I strongly disagree” to +3 =“I strongly agree”.

**Table 1:** Examples of illustrative quotes reported by Ventura et al. [31] and the related, balanced quantitative arguments developed for the current study for each of six main themes.

Main theme: Subtheme	Original Quote	Arguments in the questionnaire
**Cow and calf emotions**: Bond between cow and calf	“The cow being a mother is supposed to have an emotional string attached to her calf….by no means having a lesser degree of recognition compared to a human mother” (p. 6109)	1. The cow has an emotional attachment to her calf.
		2. The attachment between cow and calf cannot be compared to that between human mother and child.
**Calf health**: Calf nutrition	“There is no way to monitor for adequate colostrum intake if the calf nurses freely” (p. 6110) “Calves benefit from the care they receive from the cow (e.g. better access to milk and colostrum” (p. 6112). “The cow produces colostrum which isn’t commercially saleable and the calf should have free access to this for the first couple of days at least” (p. 6112)	1. By allowing calves to nurse freely from the cow, the calf has better access to milk.
		2. By separating cow and calf early, the farmer can ensure that the calf receives adequate colostrum (the first milk which is important for calf health).
**Dairy cow health and production**: Production	“Allowing the cow to be with her calf certainly keeps her happy and content. I believe a happy cow produces more milk” (p. 6112). “The farmers than have to use oxytocin on the cow to force her milk to come out….cows can get very sick if they don’t release their milk” (p. 6112)	1. A cow that is together with her calf produces more milk.
		2. If the cow nurses her calf, she won’t release her milk to the farmer.
**A natural life**: *No subthemes available*	“Nature tells us the cow is born to enjoy the companionship of her calf for a certain time and vice versa” (p. 6112). “…those calves separated early from their mothers are believed to have low socialization and [are] more stressed when mixed later with groupmates in a pen.” (p. 6112)	1. Nature tells us the cow enjoys the companionship of her calf for a certain time.
		2. Even when separated early from the cow, calves can develop normal social behavior.
**Dissatisfaction with industry motives**: Wrong focus, profit motive	“I think early cow-calf separation is practiced for the purpose of reducing labor management of the owner, not for the cow-calf wellbeing” (p. 6113). “Why can’t farmers treat the very thing that makes them so much money with some respect?” (p. 6113)	1. Early cow-calf separation is done to reduce labor for the farmer and does not consider the welfare of the calf and cow.
		2. Farmers treat their cows with respect because cows are their livelihood.
**Changeability of dairy farming structure:** *No subthemes available*	"If most of the problems around leaving the cow and calf together are a matter of environment, why not change the environment?" (p. 6113)	1. Farms that separate cows and calves early cannot easily change practices.
		2. Housing systems on dairy farms can be changed to maximize benefits for cows and calves.

Before participants were presented the different arguments they received the following information as background on the topic:

“On dairy farms cows are kept for the purpose of producing milk for human consumption. In order to produce milk, a cow needs to give birth to a calf approximately once a year. Once born, there are two main ways calves can be handled:

1. *The calf is separated from the cow within the first few hours of birth and moved to a separate barn so that there is no contact between cow and calf (“early separation”)*.
  OR
2. *The calf stays with the cow for some days or weeks and is then moved to a separate barn and there is no contact between cow and calf from then on (“later separation”)*.*”*

The two options regarding the timing of when the calf was removed from its mother, early or later separation, were randomly presented to control for order effects. Immediately following the provision of the background information participants were asked to give their opinion on when calves should be separated from the cows using a 7-point scale ranging from -3 = “They definitely should be separated later” to 0 = “I am not sure” to +3 = “They definitely should be separated early”. The question was then repeated after participants were presented with the 22 arguments outlined in Table 1 to test for shifts in opinion caused by the information provided through the arguments.

Additional survey questions asked included peoples’ value orientation on a Short Schwartz Value Scale (SSVS) [42], belief in animal mind following Hills [43], pet ownership/pet relations using the pet relation scale by Kafer et al. [44], familiarity with agriculture, dietary convictions and demographics such as gender, age, having children and breastfeeding own children to further describe the different citizen segments.

To control if participants read the questions we included two types of quality check. First, a quality check question was randomly positioned within the arguments that simply asked participants to tick “I agree” to control if they really read the questions. Second, participants that answered the survey in less than 1/3 of the average response time were excluded, as we assumed that answering the questionnaire in this little time precluded a thorough reading of all questions.

### Participant recruitment and data collection

Participants in both countries were recruited online in June and July 2015 through an external provider. For the US sample, Amazon’s Mechanical Turk platform was used to invite participants to take part in the study. In Germany, participants were recruited via Clickworker, a platform similar to Mechanical Turk, but focused exclusively on German residents. Crowdsourcing platforms such as Amazon’s Mechanical Turk and Clickworker provide access to a large and diverse subject pool at comparably low costs compared to the more traditional face to face or telephone interviews which helps to fasten empirical experiments and theory validation in research [45]. Although participants recruited through crowdsourcing platforms differ from the general public due to possible self-selection biases, they have been shown to be more diverse than student samples and importantly produce reliable outcomes comparable to offline samples [45,46]. Before taking part in the survey, participants were briefly informed about the topic of the survey and were required to give consent to take part. All participants were free to exit the survey at any time. The reward given to those respondents that completed the survey was US$1 or 1€ in the US and German sample, respectively.

A total of 517 participants from the US and 601 from Germany took part in the study. After removing 147 participants that failed the quality check question, completed the survey in less than 1/3 of the average response time or did not answer all questions in the main part of the study, the responses from 967 participants (476 US and 491 German participants) remained for further analyses.

### Data analyses

The data were analyzed using IBM SPSS Statistics 23. We initially undertook an exploratory factor analysis (see [47]) in which we included all of the arguments presented by the participants on cow-calf separation. This process allowed us to identify relations between single items and to increase reliability of the measurement by creating scales that could be used for further analyses. Variables showing only low (<0.4) factor loadings or high (≥0.4) factor loadings on more than one factor were excluded from analysis. Three reliable factors were identified, which included 16 of the 22 arguments (Kayser Meyer Olkin criterion = 0.88; Bartlett’s test of sphericity: p = 0.000; total variance explained = 54.74%). The first factor (Cronbachs Alpha = 0.77) primarily included arguments that focused on the emotional life of the cow and calf. In addition to those, an argument referring to changing housing systems to benefit the animals was also included due to high factor loading. Factor 2 (Cronbachs Alpha = 0.74) reflected arguments that were in favor of early separation; whereby, health, emotion and management arguments were mixed together. Factor 3 (Cronbachs Alpha = 0.68) related to arguments in favor of later separation that were focused on health, production and management.

Using these three factors as well as the question concerning the right time of separation (after the provision of arguments), we undertook a cluster analyses to identify different segments within the sample populations that were reasonably homogenous within each segment but as heterogeneous as possible between the segments [48]. This procedure was divided into three parts. First, outliers in the sample were identified using Single Linkage clustering method. Nine participants were identified as outliers and excluded from further analyses. Second, the optimal number of clusters and the cluster means were determined using the Ward Method [49]. Thirdly, a partitioning K-Means cluster analysis (suitable for larger sample sizes (> 500 cases [50])) was done to form the clusters. This method forms random clusters based on starting points identified with the Ward Method at the outset and reassigns participants with the aim of minimizing within cluster variation to create homogeneous groups. This process resulted in the identification of a three-cluster solution for the present study. Using discriminate analysis 97.8% of cases could be classified correctly, indicating a good cluster solution.

To further characterize the clusters and test for differences between clusters, one-way Analysis of Variance (ANOVA) with post-hoc tests, independent T-tests, paired-samples T-tests with correlations and cross tabulation with chi-square and z-tests were used. To create reliable scales for cluster comparison, two further factor analyses were conducted: firstly, participant’s belief in animal mind with two reliable factors: a) Belief in animal mind negative and b) Belief in animal mind positive; Kayser Meyer Olkin criterion = 0.73; Bartlett’s test of sphericity: p = 0.000; total variance explained = 54.1%). Secondly, the pet relation scale was calculated (one reliable factor; Kayser Meyer Olkin = 0.89; Bartlett’s test of sphericity: p = 0.000; total variance explained = 61.0%). To assess the more general values of respondents, two constructs from the SSVS (‘conservation’ and ‘self-transcendence’) were calculated as proposed by Lindeman and Verkasalo [42]. In this case, conservation measures “whether people resist change and emphasize self-restriction and order or whether they are ready for new experiences and emphasize independent action and thought” and self-transcendence measures peoples’ motivation “to transcend selfish concerns and promote the welfare of others or whether they are more motivated to enhance their own personal interests even at the expense of others.” [42].

## Results

### Sample description

Both the US and German samples were convenience samples. No quotas for demographic requirements were set during the survey. The distribution of gender, age and region compared to census data for both populations is shown in Table 2. With regard to age, both samples were young relative to the census data. The regional distribution in the US sample was similar to the population data but for Germany participants from the western part were overrepresented.

**Table 2:** Distribution of demographics in the US (n = 476) and German (n = 491) online sample compared to census data from both countries.

Attribute	US sample	US population^a^^,^^b^	German sample	German population^c^^,^ ^d^^,^ ^e^
**Gender**				
Male	49.4%	49.2%	50.7%	49.0%
Female	50.6%	50.8%	48.3%	51.0%
No specification	-	-	1.0%	-
**Age**				
18–29	39.9%	21.8%	39.3%	16.7%
30–39	31.9%	16.9%	25.3%	14.4%
40–49	16.6%	16.9%	14.7%	18.3%
50–59	8.8%	18.0%	15.7%	18.3%
> 60	2.7%	26.4%	5.1%	32.3%
**Region USA/Germany**				
Northeast/North	20.0%	17.6%	17.1%	18.1%
Midwest/ East	22.3%	21.2%	27.1%	30.4%
South/South	38.2%	37.6%	22.4%	29.8%
West/West	19.5%	23.6%	33.4%	21.7%

^a^ Gender and age [51]

^b^ Region [52]

^c^ Gender [53]

^d^ Age [54]

^e^ Region [55]

To test participants’ familiarity with dairy farming they were asked to state their frequency of visits to commercial dairy farms; 55% and 56% in the US and German sample stated that they have never been on a commercial dairy farm, 21.6% and 21.8% reported having visited a dairy farm once, 21.8% and 20.8% a few times and only 1.5% and 1.4% reported having regularly visited a commercial dairy farm, respectively.

### Favoring early or later separation

After reading the information on cow-calf separation, participants were asked to indicate their opinion on whether separation should occur early or later. The question was repeated a second time after participants had the opportunity to read the 22 arguments presented on this topic (see Table 1). For the US sample more than half of respondents were initially in favor of later separation (55.1%), 17.6% were unsure and 27.3% favored early separation. In contrast, 68.7% of Germans favored later separation, 11.2% were unsure and 20.2% favored early separation before the presentation of arguments (Table 3).

**Table 3:** Responses by American (US; n = 476) and German (German; n = 491) participants’ to whether cow-calf separation should take place early or later, BEFORE and AFTER the provision of arguments.

	They definitely should be separated later.	They should be separated later.	They probably should be separated later.	I am not sure.	They probably should be separated early.	They should be separated early.	They definitely should be separated early.
US BEFORE	20.6%	17.9%	16.6%	17.6%	14.9%	6.9%	5.5%
US AFTER	18.5%	18.5%	19.5%	21.8%	12.2%	5.9%	3.6%
German BEFORE	31.6%	21.0%	16.1%	11.2%	9.4%	6.3%	4.5%
German AFTER	23.8%	21.8%	21.4%	18.5%	7.7%	4.3%	2.4%

Regardless of country, we noted an increase in the total number of unsure responses and a decline in the responses at the extreme ends of the scales for later separation, as well as a decline for all categories favoring early separation, after participants were provided the 22 different arguments for and against early and late separation.

### Attitudes towards cow-calf separation—Description of the clusters

The cluster analysis, based on the three factors that emerged once the participants responded to the different arguments for early and later separation and on the question of when cows and calves should be separated, identified three unique groups of participants. The three clusters were named “Late”, “Unsure”, and “Early”, referring to the preferred time of separation. Participants from the US and German samples were found in each of the three clusters although proportions varied to some degrees. For example, the proportion of US residents was higher in the “Early” cluster compared to the German but this was reversed for the “Late” cluster; with the “Unsure” cluster having approximately equal proportions of each country.

#### Cluster “Late”–Favor later separation

This cluster represented 370 participants (38.7% of the entire sample) making it intermediate in size relative to the other two clusters. Respondents in this cluster were strongly in favor of later separation of cow and calf (Table 4). After the provision of arguments there was an increase in the support for later separation, especially at the middle of the scale supporting later separation, while answers of respondents showing extreme support before remained for the most part unaffected after the provision of the arguments.

**Table 4:** Percentages and means (± S.D.) falling into the 7-point Likert scale on whether cow-calf separation should take place early or later BEFORE and AFTER the provision of arguments.

	-3	-2	-1	0	+1	+2	+3	Mean (Standard deviation)	t-value
Cluster “Late” BEFORE	55.9%	29.7%	10.5%	2.4%	0.3%	0.8%	0.3%	-2.35 (0.93)	3.59***
Cluster “Late” AFTER	54.6%	42.4%	2.4%	0.5%	-	-	-	-2.52 (0.58)	
Cluster “Unsure” BEFORE	9.1%	17.7%	26.4%	27.3%	12.5%	4.1%	2.9%	-0.60 (1.41)	0.56^n.s.^
Cluster “Unsure” AFTER	-	9.1%	45.3%	45.6%	-	-	-	-0.64 (0.65)	
Cluster “Early” BEFORE	2.3%	2.3%	5.3%	8.8%	36.8%	25.7%	18.7%	1.27 (1.35)	-3.34**
Cluster “Early” AFTER	-	-	-	0.6%	55.6%	28.7%	15.2%	1.58 (0.75)	

Responses are shown separately BEFORE and AFTER the provision of arguments, together with the p-value from a paired-sample t-tests comparing these responses. Responses are also shown separately for the “Late”, “Unsure”, and “Early” clusters. Scales: Separation question: 7-point scale from -3 =“They definitely should be separated later”, -2 =“They should be separated later”, -1 =“The probably should be separated later”, 0 =“I am not sure”, +1 =“The probably should be separated early”, +2 =“They should be separated early”, +3 =“They definitely should be separated early”. Cluster “Late” =“Favor later separation”; cluster “Unsure” =“Unsure whether to favor later separation”; cluster “Early” =“Favor early separation”.

***p≤0.001

**p≤0.01

n.s.: non-significant

We also considered how many participants changed their responses after the provision of different arguments. In the”Late” cluster, 71.1% of the participants did not change their ‘before’ response, 14.6% of participants strengthened their existing opinion to support later separation and 10.5% chose to weaken their support for late separation practice.

Table 5 displays the responses to the variables used to build the clusters. Cluster “Late” participants showed high agreement with arguments that pertained to the animals’ emotions in Factor 1 such as “The cow has an emotional attachment to her calf.” Respondents within this cluster rejected the argument that the attachment between cow and calf differs from that of a human mother and child. Compared to the other clusters, participants in this cluster disagreed with arguments in favor of potential health and emotional advantages associated with early separation (Factor 2). Furthermore, participants believed that it would not be hard to change practices on farms that currently separate early. This cluster of participants agreed with arguments that promoted potential health and production benefits of later separation (Factor 3) and assumed that early separation was done to reduce labor for the farmer and did not consider the welfare of the cow and the calf.

**Table 5:** Cluster “Late”, “Unsure” and “Early” means (±S.D.) for the cluster-building variables “When do you think calves should be separated from the cow?” and factor 1, 2 and 3 arising from exploratory factor analysis.

Item (Factor loading)	Cluster		
	“Late” n = 370	“Unsure” n = 417	“Early” n = 171
^2^^**;**^^3^When do you think calves should be separated from the cow?***	-2.51^b^^,^^c^ (0.58)	-0.64^a^^,^^c^ (0.64)	1.58^a^^,^^b^ (0.75)
^1^Factor 1: Considerations of the emotional life of cows and calves on farms***, CA = 0.77	1.72^b^^,^^c^ (0.80)	0.87^a^ (0.77)	0.75^a^ (0.91)
^2^The cow has an emotional attachment to her calf.(0.77)	2.03 (0.97)	1.28 (1.07)	1.11 (1.27)
^2^Cows and calves enjoy being together. (0.70)	2.12 (0.89)	1.24 (1.01)	1.12 (1.12)
^2^Cows and calves experience few emotions.(-0.69)	-1.65 (1.20)	-0.68 (1.25)	-0.47 (1.44)
^2^The attachment between cow and calf is different from that between human mother and child. (-0.68)	-0.97 (1.63)	0.00 (1.38)	0.08 (1.59)
^1^Housing systems on dairy farms can be changed to maximize benefits for cows and calves. (0.49)	1.81 (1.08)	1.13 (1.03)	1.12 (1.08)
^2^Factor 2: Arguments favoring early separation: health, emotion and management***, CA = 0.74	-1.08^b^^,^^c^ (0.82)	-0.15^a^^,^^c^ (0.64)	0.37 (0.75)
^2^Early separation reduces disease transmission from the cow to the calf. (0.70)	-0.98 (1.36)	0.08 (1.16)	0.40 (1.23)
^2^With later separation there is a risk that the cow injures the calf. (0.70)	-1.44 (1.21)	-0.48 (1.13)	-0.07 (1.38)
^2^It is better for cow and calf to separate early because later separation is very hard on the mother. (0.65)	-1.00 (1.32)	0.16 (1.23)	1.32 (1.36)
^1^The calf’s suckling can damage the cows’ teats. (0.64)	-1.40 (1.26)	-0.59 (1.19)	-0.18 (1.38)
^2^By separating cow and calf early, the farmer can ensure that the calf receives adequate colostrum (the first milk which is important for calf health).(0.58)	-0.88 (1.55)	0.06 (1.19)	0.53 (1.29)
^2^For farms that separate cows and calves early it is hard to change practices. (0.42)	-0.78 (1.43)	-0.11 (1.17)	0.19 (1.33)
^2^Factor 3: Arguments favoring later separation: health, production and management***, CA = 0.68	1.37^b^^,^^c^ (0.70)	0.59^a^^,^^c^ (0.60)	0.34^a^^,^^b^ (0.82)
^2^A cow that is together with her calf produces more milk. (0.65)	1.14 (1.08)	0.47 (0.93)	0.20 (1.22)
^2^Frequent suckling by the calf helps prevent common cow diseases. (0.64)	1.18 (1.10)	0.41 (0.90)	0.35 (1.06)
^2^Early separation of cow and calf does not improve calf health. (0.59)	1.35 (1.57)	0.45 (1.12)	0.30 (1.27)
^2^Early cow-calf separation is done to reduce labor for the farmer and does not consider the welfare of the calf and cow. (0.59)	1.28 (1.41)	0.49 (1.09)	0.16 (1.27)
^2^By allowing calves to nurse freely from the cow, the calf has better access to milk. (0.57)	1.92 (1.11)	1.14 (1.05)	0.68 (1.36)

The table shows a mean comparison of all four cluster-building variables and all single variables using ANOVA and post-hoc tests. Cluster “Late” =“Favor later separation”; cluster “Unsure” =“Unsure whether to favor later separation”; cluster “Early” =“Favor early separation”. CA = Cronbach’s Alpha for the factor. Numbers in brackets behind the single items indicate loadings on the factor above. Numbers in the main part of table show means and standard deviation in brackets. Means for the factors show non-weighted factor scores. Scales: Separation question: 7-point scale from -3 =“They definitely should be separated later” over 0 =“I am not sure” to +3 =“They definitely should be separated early”; all other questions: 7-point scale from -3 =“I strongly disagree” over 0 =“I neither agree nor disagree” to +3 =“I strongly agree”. Stars indicate differences with ***p≤0.000;

^a, b, c^ = Letters indicate significant differences (p≤0.05) between clusters according to post-hoc tests, e.g. a indicates that this cluster differs from cluster”Late” in this variable with p≤0.05.

^1^ = LSD post-hoc test was used because of no differences in variances in clusters.

^2^ = Tamhane post-hoc test was used because of differences in variances in clusters.

^3^Separation question asked after the presentation of 22 different arguments.

Table 6 shows arguments for early and later separation that could not be incorporated into the factors due to either low factor scores (<0.4) or due to high scores on more than one factor (>0.4) (see [47]). Table 7 compares factors for belief in animal mind, the pet relation scale, and the two constructs arising from the SSVS. Cluster “Late” differed from the other clusters in most of the statements. These participants, for example, related early separation with distress for the cow and calf and believed that the cow and calf enjoyed mutual companionship. They also disagreed with the argument that by nursing a calf, the cow will not release her milk to the farmer and were skeptical regarding the calves’ ability to develop normal social behavior when separated early from their mothers. Cluster “Late” also had the highest belief in the cow’s mind, i.e. they agreed that cows are conscious and aware of what is happening to them and that they are capable of experiencing feelings and emotions. In contrast, this cluster was more indecisive in their responses to whether cows were more likely to respond to instinctive urges, have limited ability to see cause and effect of an action and experience emotions less intensely than humans.

**Table 6:** Cluster “Late”, “Unsure” and “Early” means (±S.D.) for cluster-describing variables about weaning that were not related to the factors.

Item (factor loading)	Cluster		
	“Late” n = 370	“Unsure” n = 417	“Early” n = 171
**Cow-calf separation arguments not included in the factors**			
Early separation of cow and calf causes distress for both.***	1.89^b^^,^^c^ (1.16)	0.85^a^^,^^c^ (1.10)	0.16^a^^,^^b^ (1.49)
The cow helps to protect the calf from threats and injury.***	1.89^b^^,^^c^ (0.96)	1.12^a^^,^^c^ (1.00)	0.72^a^^,^^b^ (1.44)
If the cow nurses her calf, she won’t release her milk to the farmer.***	-0.44^b^^,^^c^ (1.55)	-0.15^a^^,^^c^ (1.16)	0.32^a^^,^^b^ (1.43)
Nature tells us the cow enjoys the companionship of her calf for a certain time.***	1.82^b^^,^^c^ (0.95)	1.01^a^ (0.93)	0.91^a^ (1.11)
Even when separated early from the cow, calves can develop normal social behavior.***	-0.25^b^^,^^c^ (1.33)	0.33^a^^,^^c^ (0.96)	0.84^a^^,^^b^ (1.19)
Farmers treat their cows with respect because cows are their livelihood.***	0.20^c^ (1.59)	0.42^c^ (1.24)	0.73^a^^,^^b^ (1.43)

Displayed are mean comparisons using ANOVA and post-hoc tests for the arguments not included as cluster-building variables. Cluster “Late” =“Favor later separation”; cluster “Unsure” =“Unsure whether to favor later separation”; cluster “Early” =“Favor early separation”. Differences between clusters were tested using ANOVA and post-hoc tests. Stars indicate differences according to ANOVA with ***p≤0.000. Tamhane post-hoc test was used because of differences in variances in clusters.

^a, b, c^ = Letters indicate significant differences (p≤0.05) between clusters according to post-hoc tests, e.g. ^a^ indicates that this cluster differs from cluster “Late” in this variable with p≤0.05.

**Table 7:** Cluster “Late“, “Unsure”and “Early” means (±S.D.) for cluster describing variables in relation to pets and Schwartz values.

Item (factor loading)	Cluster		
	“Late” n = 370	“Unsure” n = 417	“Early” n = 171
^1^ Factor: Belief in animal mind positive***, CA = 0.65	1.27^b^^,^^c^ (0.98)	0.86^a^^,^^c^ (0.99)	0.68^a^^,^^b^ (1.05)
^1^Cows are conscious and aware of what is happening to them. (0.80)	1.22 (1.44)	0.85 (1.44)	0.71 (1.38)
^1^Cows are able to think to some extent to solve problems and make decisions about what to do. (0.75)	0.58 (1.49)	0.23 (1.40)	0.06 (1.43)
^2^Cows are capable of experiencing a range of feelings and emotions (e.g., pain, suffering, contentment, maternal affection, aggression, fear, frustration, loneliness, and boredom). (0.68)	2.02 (1.06)	1.50 (1.12)	1.27 (1.32)
^2^ Factor: Belief in animal mind negative***, CA = 0.62	0.08^b^^,^^c^ (1.22)	0.42^a^ (1.00)	0.62^a^ (1.10)
^2^Cows have limited mental ability to see cause and effect of an action.(0.87)	0.24 (1.49)	0.52 (1.38)	0.73 (1.30)
^2^Cows experience emotions less intensely than humans. (0.72)	-0.18 (1–61)	0.18 (1.37)	0.57 (1.36)
^2^Cows are more mechanically responding to instinctive urges without awareness of what they are doing. (0.56)	0.18 (1.62)	0.57 (1.34)	0.55 (1.30)
Factor: Pet relation scale***, CA = 0.87	0.64^b^^,^^c^ (1.34)	0.27^a^ (1.34)	0.26^a^ (1.31)
^1^In many ways, my pet is the best friend I have. (0.87)	0.80 (1.79)	0.28 (1.79)	0.20 (1.73)
^1^My pet is an equal in my family. (0.83)	1.46 (1.52)	0.97 (1.66)	0.96 (1.61)
^1^My pet gives me a reason for getting up in the morning. (0.82)	0.78 (1.70)	0.34 (1.73)	0.25 (1.73)
^1^My pet is constantly at my side. (0.82)	1.01 (1.69)	0.53 (1.68)	0.62 (1.66)
^1^I talk to my pet about things that bother me. (0.75)	0.04 (1.90)	-0.30 (1.78)	-0.29 (1.82)
^1^Making me laugh is part of my pet’s job. (0.56)	-0.26^.^ (1.79)	-0.18^.^(1.68)	-0.18 (1.73)
**Schwartz Values**			
^1^Conservation^n.s.^	0.42^n.s.^ (1.24)	0.40 ^n.s^ (1.19)	0.58 ^n.s^ (1.12)
^1^Self-Transcendence***	-0.28^c^ (0.98)	-0.41^c^ (0.99)	-0.80^a^^,^^b^ (1.04)

Displayed are mean comparisons using ANOVA and post-hoc tests for factors indicating participants’ “Belief in animal mind”, their pet relation and their value orientation measured on a Short Schwarz Value Scale. Cluster “Late” =“Favor later separation”; cluster “Unsure” =“Unsure whether to favor later separation”; cluster “Early” =“Favor early separation”. CA = Cronbach’s Alpha for the factors. Numbers in brackets behind the items indicate loadings on the factor above. Numbers in the table show means and standard deviation in brackets. Scales: Schwartz values: 7-point scale from: -1 =“Opposed to my principles” over “0 =“not important” to +5 =“Of surpreme importance; all other questions: 7-point scale from -3 =“I strongly disagree” over 0 =“I neither agree nor disagree” to +3 =“I strongly agree”. Stars indicate differences according to ANOVA with ***p≤0.000. n.s. = indicates no differences.

^1^ = LSD post-hoc test was used because of no differences in variances in clusters.

^2^ = Tamhane post-hoc test was used because of differences in variances in clusters.

^a, b, c^ = Letters indicate significant differences (p≤0.05) between clusters according to post-hoc tests, e.g. a indicates that this cluster differs from cluster “Late” in this variable with p≤0.05;

n.s. = indicates no differences compared to the other cluster (p≥0.05).

In cluster “Late”, the proportion of participants having children was higher compared to the other two clusters (“Late” = 51.6%, “Unsure” = 32.6%, “Early” = 27.5%, p≤0.05), but no differences were observed in the proportion reporting breastfeeding (“Late” = 77.0%, “Unsure” = 73.3%, “Early” = 76.6%, p˃0.05). With regard to pet ownership, cluster “Late” showed the highest proportion, differing from clusters”Unsure” and “Early” (”Late” = 59.7%, “Unsure” = 49.4%, “Early” = 46.2%, p≤0.05). Pet owner participants in cluster “Late” showed the highest score on the pet relation scale indicating a close relationship to their pet; their pet gave them a reason to get up in the morning, was the best friend they have and was constantly at their side. In terms of general values, cluster “Late” differed in the construct of self-transcendence from cluster “Early”, showing the highest score of all groups (see Table 6). Clusters did not differ in frequency of having visited a commercial dairy farm (p˃0.05).

In Table 8, demographic characteristics of the clusters are shown. Cluster “Late” also had a higher proportion of German participants and the highest share of female participants. Cluster “Late” contained fewer participants under the age of 29 years compared to the other two clusters and also had a higher share of older participants. The proportion of vegetarians was higher in this cluster and the number of participants within this cluster that have a family member working in agriculture was lower. The share of participants having regular contact with agriculture through others such as friends or neighbors was higher. No differences between the clusters were found with regard to whether participants lived in rural or urban areas.

**Table 8:** Percentage of participants falling into clusters “Late”, “Unsure” and “Early”, in relation to participant demographics.

	Cluster		
	“Late”	“Unsure”	“Early”
Total participants	n = 370 = 100%	n = 417 = 100%	n = 171 = 100%
Country**			
USA	43.0% ^c^	50.4% ^n.s.^	59.1% ^a^
Germany	57.0% ^c^	49.6% ^n.s.^	40.9% ^a^
Sex***			
Female	62.2% ^b^^,^^c^	46.3% ^a^^,^^c^	31.6% ^a^^,^^b^
Male	37.3% ^b^^,^^c^	53.0% ^a^^,^^c^	68.4% ^a^^,^^b^
No answer	0.5% ^n.s.^	0.7% ^n.s.^	0.0% ^n.s.^
Age***			
18–29 years	30.3% ^b^^,^^c^	42.7% ^a^	52.6% ^a^
30–39 years	29.2% ^n.s.^	28.3% ^n.s.^	27.5% ^n.s.^
40–49 years	18.6%^c^	15.3% ^n.s.^	8.8% ^a^
50–59 years	16.8% ^b^	9.8% ^a^	9.4% ^n.s.^
>60 years	5.1% ^n.s.^	3.8% ^n.s.^	1.8% ^n.s.^
**Residency** ^**n.s.**^			
Urban	32.2% ^n.s.^	31.4% ^n.s.^	34.5% ^n.s.^
Suburban	44.3% ^n.s.^	46.5% ^n.s.^	44.4% ^n.s.^
Rural (not on a farm)	21.9% ^n.s.^	20.4% ^n.s.^	20.5% ^n.s.^
Rural (on a farm)	1.6% ^n.s.^	1.7% ^n.s.^	0.6% ^n.s.^
Diet***			
No restrictions	86.5% ^b^^,^^c^	92.1% ^a^	94.2% ^a^
No Meat but fish	2.4% ^n.s.^	3.8% ^n.s.^	4.1% ^n.s.^
Vegetarian	7.3% ^b^^,^^c^	3.4% ^a^	1.2% ^a^
Vegan	3.8% ^b^	0.7% ^a^	0.6% ^n.s.^
**Connection to agriculture (multiple answers possible)**			
None	30.0%^n.s.^	35.5% ^n.s.^	36.8% ^n.s.^
Spent holidays on farms	29.5% ^n.s.^	24.4% ^n.s.^	25.7% ^n.s.^
Seen pictures and movies about farms	54.1% ^n.s.^	51.1% ^n.s.^	49.1% ^n.s.^
Are regularly in contact with agriculture through others	29.5% ^b^	19.9% ^a^	22.8% ^n.s.^
Someone in the family works in agriculture	13.8% ^c^	18.2% ^n.s.^	22.2% ^a^
Work/Worked themselves in agriculture	8.4% ^n.s.^	6.2% ^n.s.^	9.9% ^n.s.^

Cluster “Late” =“Favor later separation”; cluster “Unsure” =“Unsure whether to favor later separation”; cluster “Early” =“Favor early separation”. Differences between clusters were tested using Chi-square test and Cross tabulation z-test with Bonferroni adjustment of p-levels. Stars indicate differences according to Chi-Square Test with ***p≤0.000 and **p≤0.01. ^n.s.^ = indicates no differences.

^a, b, c^ = Letters indicate significant differences (p≤0.05) between clusters according to z-test in the cross tabulation, e.g. ^a^ indicates that this cluster differs from cluster “Late” in this variable with p≤0.05. ^n.s.^ = indicates no differences compared to the other cluster (p≥0.05).

#### Cluster “Unsure”–Unsure of whether to favor later separation

The “Unsure” cluster was the largest of the three clusters and included just under half of all of the participants (43.6%). This cluster also favored later separation but showed less certainty. After seeing the arguments, 42.1% of participants maintained their initial opinion on the separation question. Of the 57.9% who changed their opinion, 16.8% strengthening their support in favor of later separation and 25.9% either changed their initial support in favor of early or late separation to an unsure response. The means in this cluster did not change from before to after having seen the arguments.

The participants in the “Unsure” cluster showed lower agreement to arguments highlighting the emotions of cow and calf (Factor 1) compared to the”Late” cluster, but still agreed that the cow has an emotional attachment to her calf (see Table 5). This cluster rejected, although to a lower extent than cluster”Late”, that cows and calves experience few emotions. However, they were also unsure about whether the attachment between cow and calf is different from that between a human mother and child and they agreed that housing systems on dairy farms can be changed to maximize benefits for the animals, but to a lesser extent compared to cluster “Late”.

The “Unsure” cluster differed from the two other clusters regarding their responses to the arguments presented in favor of potential health and emotional advantages associated with early separation (Factor 2). Cluster “Unsure” respondents were uncertain whether to agree or disagree with the health and emotional arguments presented in this factor. Only in the arguments about the cow being able to injure the calf or the calves suckling damaging the cows’ teats did these participants show disagreement. With regard to health, production and management arguments favoring later separation (Factor 3), “Unsure” participants showed some support for each of these arguments (differing from the “Late” cluster in all cases). The “Unsure” cluster was intermediate in terms of support of the argument that a calf has better access to milk if it can nurse freely from the cow compared to the “Late” and “Early” cluster.

Participants within the “Unsure” cluster agreed that early separation causes distress for the animals and that the cow protects the calf from threats and injuries. This cluster also agreed with the arguments that were in accordance with the nature of the cow and calf to enjoy companionship for a while. They were, however, unsure if the cow releases her milk to the farmer if kept with her calf and tended to be unsure if calves can develop normal social behavior. They also tended to agree that farmers treat their cows with respect because they are their livelihood. Cluster “Unsure” could also be characterized as having some belief in animal mind, differing in this respect from the “Late” and to some extent from the “Early” cluster. Participants in this cluster agreed that cows are conscious and aware of what is happening to them and that they are able to experience emotions. They were however unsure if cows are able to solve problems and make decisions. They agreed that cows respond more mechanically to instinctive urges and that they have limited ability to see cause and effect. They also tended to agree that cows experience emotions less intensely than humans. Participants in this cluster scored lower on the pet relation scale than the “Late” cluster, and were similar to the “Early” cluster in this respect. Regarding the SSVS, the “Unsure” cluster differed only from the “Early” cluster in the construct of self-transcendence.

In the “Unsure” cluster, the US and German participants were equally distributed. There were slightly more men than women in this cluster. A higher proportion of participants were less than 29 years of age in this cluster compared to the”Late” cluster, and there were fewer vegetarians. There were also fewer participants in regular contact with agriculture through others such as friends or neighbors compared to the “Late” cluster. The percentage of people having children or pets was lower in this cluster compared to the “Late” cluster but did not differ from the “Early” cluster.

#### Cluster “Early”–Favor early separation

This cluster was the smallest with participants’ representing approximately 18% of the entire sample. Participants in this cluster favored early separation of the cow and calf in dairy farming. Within this cluster there were fewer unsure answers and support for early separation increased after reading the different arguments (see Table 4). In this cluster, 55% of participants did not change their response to the separation question after the arguments were presented, whereas 17.5% weakened and 9.4% strengthened their existing opinion to support early separation. Within this cluster 9.9% of participants favored later separation before the arguments and changed their opinion to favor late separation afterwards. Regarding the arguments in Factor 1, this cluster only differed from the”Late” cluster. With regard to Factor 2, this cluster showed the highest agreement compared to the other two clusters. Participants showed higher agreements with arguments favoring early separation, particularly with respect to the argument that later separation is hard on the mother. The participants in this cluster appeared unsure regarding arguments about calf injuries and teat damage presented in favor of later separation, focusing rather on the farmer being able to ensure adequate colostrum intake with early separation. Participants in this cluster also tended to agree that it is hard for farms to change practices associated with early separation.

Cluster “Early” had the lowest means in Factor 3 compared to the other two clusters. They were also unsure if a cow produces more milk when together with her calf and if frequent suckling helps prevent cow diseases. They were also unsure if early separation improves calf health and whether it reduces labor. They did agree, albeit to a lesser extent, that calves have better access to milk if kept together with the cow.

Cluster “Early” participants were unsure if early separation causes distress for the animals but they did agree (albeit to a lesser extent than the other two clusters) that the cow helps to protect the calf from threats and injury. Participants within this cluster tended to agree that the cow would not release her milk to the farmer if kept together with her calf. This cluster agreed that calves can develop normal social behavior even when separated and that farmers treat their cows with respect. Although this cluster showed the lowest belief in animal mind, they did think that cows were able to experience emotions but did so less intensely than humans. This cluster showed the same pet relation score as cluster “Unsure”. In terms of Schwartz-Values, cluster “Early” showed the lowest score on self-transcendence.

Cluster “Early” contained a higher share of US participants. The majority of participants represented in this cluster were men and half of the participants were under the age of 29. The proportion of vegetarians in this cluster was the lowest of all three clusters. Similar to the “Unsure” cluster approximately 23% of the participants in this cluster had family members working in agriculture. The number of participants having children was the lowest in this cluster, and differed from the”Late” cluster. The proportion of participants reporting that they were current pet owners was also less compared to the”Late” cluster.

## Discussion

This study used a quantitative approach to provide insights into citizen attitudes towards separation of cow and calf. The online survey was cost and time efficient compared to other data collection methods. Additionally, it helped avoid social desirability biases [56]. Possible disadvantages of this methodology are that the number of indecisive answers is higher in online surveys compared to face-to-face interviews [56]. To mitigate the effects of these challenges, we used 7-point scales instead of 5-point scales, as the potential for using the mid-point category decreases when there are more scale steps [57]. Demographics such as sex and region were close to census data but both the U.S. and German samples were younger compared to the original populations. Also, given that we used a convenience sample, our results should not be considered representative on a national scale. However, our work brings to light the contentiousness of cow-calf separation and can be used as basis for future quantitative studies that make use of representative samples.

### Demographic differences between the clusters

We found few differences in demographics between clusters. Ellis et al. [17] found differences in dairy cattle welfare perceptions between age groups, but we observed no clear pattern in the current study, although cluster “Late” showed the lowest share of young participants (between 18 and 29 years). The higher percentage of females in the “Late” cluster is in line with other studies reporting that females desire higher care for animals [5] and show higher welfare sensibility [58]. Although some work has reported that having children was associated with lower animal welfare concerns [3,36], we found no such association in our study. That more participants in cluster “Late” were parents and were female, in comparison with either cluster “Unsure” or “Early”, may be because the discussion reminded parents, particularly mothers, of their relationship with their children. We found no differences in place of residence (e.g. urban verses rural); in contrast, other studies have sometimes found that rural participants were more satisfied with farming practices [16] and trust farmers more [1]. Participants having a family member working in agriculture were more likely to support early separation in our study, perhaps because such individuals have a more positive image of farmers than those further removed from agriculture [16,59]. The increased proportion of US residents in the “Early” cluster and the increased proportion of German participants in the “Late” cluster may be a consequence of some differences in values which may be influenced by cultural norms within societies [16]. Our noted differences of participant proportions within the “Early” and “Late” clusters from the two countries suggest that the distribution of values in both societies may differ [15]. Unfortunately, our sample is not representative on either of the national scales but we encourage more work of this nature.

### Favoring early versus later separation

Irrespective of the arguments presented for and against separation, the common practice of separating cow and calf was not widely supported by either US or German participants. Boogaard et al. [16] reported that participants did not want calves to grow up without a dam. Ventura et al. [31] found more support for later separation in the general public, but that farmers and veterinarians working with cattle farmers supported early separation.

The current study was designed to present balanced arguments in favor of both early and later separation. Thus, by design, we did not expect that providing this information would shift the views of our participants. Some authors have argued that education of the public about farming practices will head off criticisms [12], but our results show that different clusters attend to different types of information and arguments. In all clusters, a relatively large share of participants kept to their initial answer. This effect, also known as confirmation bias, begins once a person has taken a position towards a topic that normally also includes justifying this position and, therefore, will preferentially favor arguments in support of their initial position [60,61]. Another explanation could be peoples’ moral intuition. Moral intuition describes a judgment without having gone through a conscious decision process but rather relies on a persons’ feeling of appropriateness [62]. For the “Late” cluster we saw a strengthening of support for later separation overall while nearly three quarters of participants kept their initial answer. For the “Unsure” cluster the sample means remained the same before and after the provision of arguments, but about one quarter of participants adopted an unsure response after having read the arguments. The notable increase of unsure responses in this cluster suggests that participants were attending to all of the arguments, and were thus confused by the intentionally balanced arguments we presented. Participants in the “Early” cluster were most engaged by the arguments in favor of early separation, and their responses stayed the same or strengthened in this regard. In summary, participants’ reaction to the provision of arguments differed between the clusters despite the provision of the same balanced arguments for and against the practice. This result suggests that efforts to educate the non-farming public will not consistently lead to the desired effects especially when the conflict is not mainly a consequence of ignorance but reflects differences in values [12]. This result is in line with a study of Ventura et al. [13], who surveyed participants before and after a farm visit. The authors found that citizens’ knowledge improved after the visit, but there was no increase in support for the farming practices (in fact, support declined).

The current study was not designed to test if opinions would shift if arguments were presented only in favor of the practice. We did not weigh the arguments on the basis of the evidence in their favor, but rather presented the most common arguments for and against cow-calf separation. Some participants may have questioned the validity of the arguments, perhaps contributing to the observed confirmation bias. Studies systematically analyzing the effects of non-balanced, evidence-based information provision would be of value, but we caution readers that in the case of cow-calf separation the evidence in favor of the various options is often weak and contradictory making an unbiased assessment of the ‘facts’ difficult.

Our results provide additional evidence in support of the assumption that people with similar animal welfare interests have similar characteristics regardless of country of origin [2]. Specifically, we found homogeneous segments in our cluster solution that included participants from both countries. Our results support the use of segmentation to better characterize people’s views on contentious topics [2,32].

### Evaluation of different arguments for later and early separation

Participants in the “Late” cluster placed great emphasis on the natural rearing process and on the emotional consequences of separation. In contrast, participants in the “Early” cluster, emphasized the disadvantages associated with later separation (e.g. distress associated with severing this bond). This latter concern is supported by the scientific literature [24] but other research has shown advantages of later separation. For example, Wagner et al. [63] found that mother-reared calves exposed to an isolation test showed lower stress levels than artificially-fed calves and were able to cope more actively with challenging situations. Another study by Wagner et al. [64], showed that calves separated later from their mothers were more interactive. Other work has found that calves reared as cow-calf pairs show better learning abilities and less food neophobia compared to individually housed calves [65].

Participants in the “Unsure” cluster seemed to be uncertain in how to evaluate the arguments. Thus, for this largest segment in our study, the information provided was either not sufficient or was (by design) too conflicting. In addition, it is possible that the two options we provided in the survey (early and later separation) did not reflect their range of preferred opinions (e.g. some may have wanted cow and calf never be separated).

### Belief in animal mind and pet relation

Our results regarding participants’ belief in animal mind are in line with those of Knight and Barnett [66] who reported that participants who perceived that the mental and emotional capabilities of animals were high, were more likely to oppose animal use. Similarly, Bastian et al. [67] found that attributing mind to an animal reduces the willingness to eat meat.

The results of our study also support the hypothesis that experiences with pets increase belief in animals’ capacities. Ellis et al. [17] found that respondents often referred to their pet when making inferences about animal emotions, and other studies found that pet owners are more concerned about animal welfare [3] and more likely to oppose animal research than non-pet owners [66]. People without a pet [30], or with less familiarity with a pet [68], perceive animals as less capable of experiencing emotions. Boogaard et al. [59] reported that pet owners perceive the quality of life of farm animals to be less positive and they also have a less positive image of farmers. Collectively, this evidence suggests that individuals with connections to companion animals are more likely to infer emotions in animals and disagree with early separation of cow and calf.

### Value orientation

Te Velde et al. [32] emphasized the importance of values in animal welfare discussions, as part of peoples’ frame of reference used to construct perceptions on various topics. Boogaard et al. [16] concluded that the acceptance of modern dairy farming is related to people’s fundamental value orientation and that socially minded respondents were more opposed to modern dairy farming practices. The scientific advisory board for agricultural policies in Germany in their report entitled “Ways towards socially accepted livestock farming”, suggested that agricultural policies must meet these values and in doing so improve the welfare for animals [69]. In the case of cow-calf separation, people favoring late separation show higher self-transcendence and therefore favoring this practice can be connected to higher empathy in people.

## Conclusions and practical implications

The majority of participants ascribed mental and emotional capabilities to cows and calves, indicating that they perceived these animals to have the capacity to experience pain and distress. Although the common practice of separating cow and calf in dairy farming after birth was not supported by the majority of participants from either the US and Germany we did note a slightly higher proportion of Germans in the cluster that favored keeping cows and calves together with the reserve being true for the cluster that favored separating cow and calf at birth. Most interestingly, the presentation of balanced arguments did not lead to overall shifts in opinions, but we did find that different clusters of participants attended to different arguments likely reflecting value differences. These results contribute to a growing body of literature indicating that educational efforts by the agricultural industries to bring public views in line with industry practices will not be successful.
